# Mortality trends of comorbid viral hepatitis C and psychoactive substance use disorders in the United States: Insights from CDC WONDER, 1999–2023

**DOI:** 10.1097/MD.0000000000049421

**Published:** 2026-06-26

**Authors:** Masira Fatima, Meraal Faisal, Warda Fatima Shafee, Mahveen Iqbal, Maliha Iqbal, Nawaal Furqan, Muddassir Khalid, Umaima Ali Usmani, Sumia Fatima, Rida Noor

**Affiliations:** a Department of Medicine, Services Institute of Medical Sciences, Lahore, Pakistan; b Department of Medicine, Foundation University, Islamabad, Pakistan; c Department of Medicine, Nishtar Medical University, Multan, Pakistan; d Department of Medicine, Continental Medical College, Lahore, Pakistan; e Department of Medicine, Saidu Medical College, Swat, Pakistan; f Department of Medicine, Rawalpindi Medical College, Rawalpindi, Pakistan; g Department of Medicine, Bolan Medical College, Quetta, Pakistan.

**Keywords:** CDC, hepatitis C, mortality trends, psychoactive substance, substance use

## Abstract

Viral hepatitis C (HCV) remains a major global health challenge, and psychoactive substance use disorders (SUD) significantly compound its burden. However, national mortality trends from comorbid HCV and psychoactive SUD are not well characterized. This study aimed to examine temporal, demographic, and regional patterns in mortality associated with comorbid HCV and psychoactive SUD in the United States. A descriptive analysis of CDC WONDER death certificate data from 1999–2023 was performed. Deaths were identified using International Statistical Classification of Diseases and Health Related Problems – 10th revision codes B17.1 and B18.2 for HCV and F10–F19 for psychoactive SUD. Age-adjusted mortality rates (AAMRs) per 100,000 persons and annual percent change (APC) with 95% confidence intervals were calculated and stratified by sex, age, race/ethnicity, U.S. geographic region, and urban-rural status. A total of 80,403 deaths occurred between 1999 and 2023. Mortality rose in 1999–2003 (APC + 16.0), declined in 2003–2009 (–2.2), increased again in 2009–2014 (+8.6), and fell after 2020 (–8.9). Males consistently had higher AAMRs, peaking at 1.6 in 2016 vs 0.6 in females. The steepest rise occurred in ages 55–74 years, while younger adults (25–44) increased after 2012. Non-Hispanic American Indians/Alaska Natives had the highest AAMRs (2.0), followed by non-Hispanic Blacks (1.2). The West and South had the greatest burden, with Colorado, Louisiana, Montana, Oklahoma, Oregon, and DC in the highest mortality decile. Rural mortality exceeded urban mortality. Comorbid HCV and psychoactive substance use disorder mortality has fluctuated over time, showing recent declines but persistent disparities by sex, race/ethnicity, and geography. Integrated prevention and treatment approaches remain urgently needed.

## 1. Introduction

In 2022, the World Health Organization declared that viral hepatitis was the second-highest viral infection fatality worldwide, with approximately 1.3 million deaths.^[1]^ It was found that 58 million people were infected with the hepatitis C virus (HCV) worldwide, and a diagnosis of 1.5 million still emerges annually. HCV causes approximately 290,000 deaths annually.^[2]^ Mental and behavioral complications from substance use also exacerbate this issue. As the World Drug Report 2023 stated, in 2021, over 296 million people had been using drugs, and 39.5 million had drug use disorders, both of which had considerably increased in the past ten years.^[3]^ In the United States, injection drug use contributes to 75% of new HCV infections, and 55.2% of the injection drug use population is living with HCV.^[4]^

HCV is a blood-borne virus and is mainly transmitted through the sharing of injection and drug use paraphernalia and blood-contaminated needles.^[5]^ Investigations have shown that over 70% of drug injectors have been diagnosed with HCV.^[6]^ Substance use disorder comorbidity with HCV results in increased liver-related and all-cause mortality, primarily due to the increased risk for reinfection and reduced adherence to treatment.^[7]^

Individuals aged 55–74 are the most affected by the HCV infection burden and have the highest mortality rates due to HCV, according to the mortality patterns by age.^[8]^ Nevertheless, the increasing death rates among the younger cohorts aged 15–34 and 35–54 are mainly driven by patterns of injectable drug use and the related risk behaviors.^[9]^ The diversity of these age brackets facilitates focused epidemiological surveillance and the implementation of specific public health initiatives.

As of now, there have been no studies specifically focused on national mortality trends related to the co-occurrence of hepatitis C and psychoactive substance use disorders (SUD). In the U.S., mortality trends in comorbid viral hepatitis C and psychoactive SUD warrant attention to inform policy and allocate resources to minimize mortality from avoidable causes. The CDC WONDER database provides extensive mortality data, facilitating comprehensive longitudinal studies. The knowledge gained can define changing patterns of epidemiology and inform the healthcare system on the need for an integrated screening, diagnosis, and treatment program for this high-risk population.

## 2. Methods

### 2.1. Study setting and population

We acquired death certificate data from the CDC WONDER (Centers for Disease Control and Prevention Wide-Ranging Online Data for Epidemiologic Research) database and studied from 1999 to 2023 for mortality related to coexisting viral hepatitis C and psychoactive SUD. Deaths were determined using the International Statistical Classification of Diseases and Related Health Problems–10^th^ Revision codes B17.1 and B18.2 for hepatitis C and F10–F19 for disorders due to psychoactive substance use. These codes have been used in previous administrative and epidemiologic studies to capture HCV and disorders due to psychoactive substance use.^[10,11]^ The dataset includes cause-of-death information from death certificates for all 50 states and the District of Columbia and has been widely employed to study national mortality trends. Multiple Cause-of-Death Public Use files were analyzed, and deaths were included if both hepatitis C and psychoactive SUD were reported anywhere on the death certificate, either as contributing or underlying causes. This study was exempt from institutional review board approval because it used de-identified, publicly available data. Reporting followed the STROBE (Strengthening the Reporting of Observational Studies in Epidemiology) guidelines

### 2.2. Data extraction

Data for population size, year of death, sex, age, race/ethnicity, state, U.S.geographic region, urban-rural classification, and place of death were abstracted from CDC WONDER. This study’s age range was from 15–34, 35–54, 55–74 and >75. Race/Ethnicity was categorized as non-Hispanic (NH) White, NH Black or African American, Hispanic or Latino, NH American Indian or Alaska Native, and NH Asian or Pacific Islander, as reported on death certificates. Place of death was classified as medical facilities (inpatient, outpatient, emergency department, death on arrival, or unknown), home, hospice, or nursing home/long-term care facility. Urban-rural classification was based on the 2013 National Center for Health Statistics Urban-Rural Classification Scheme, which defines metropolitan areas (large [≥1 million population] and medium/small [50,000–999,999]) and rural areas (<50,000). Geographic regions were categorized into Northeast, Midwest, South, and West using U.S. Census Bureau definitions.

### 2.3. Statistical analysis

To assess national trends in comorbid HCV and psychoactive substance use–related mortality, crude and age-adjusted mortality rates (AAMRs) per 100,000 population were calculated from 1999 to 2023 by year, sex, age, race/ethnicity, state, geographic region, and urban-rural status with 95% confidence intervals (CIs). Crude mortality rates were computed by dividing the number of deaths by the corresponding U.S. population for each year. AAMRs were calculated by direct age standardization to the 2000 U.S. standard population. Temporal trends were quantified using the Joinpoint Regression Program (version 5.4.0.0, National Cancer Institute), which estimates annual percent change (APC) with 95% CIs based on log-linear regression. This method identifies statistically significant changes in mortality trends over time. APC estimates were considered increasing or decreasing if the slope was significantly different from zero (two-tailed *t* test, *P* < .05)

## 3. Results

### 3.1. Overall mortality trends

**Figure 1.**
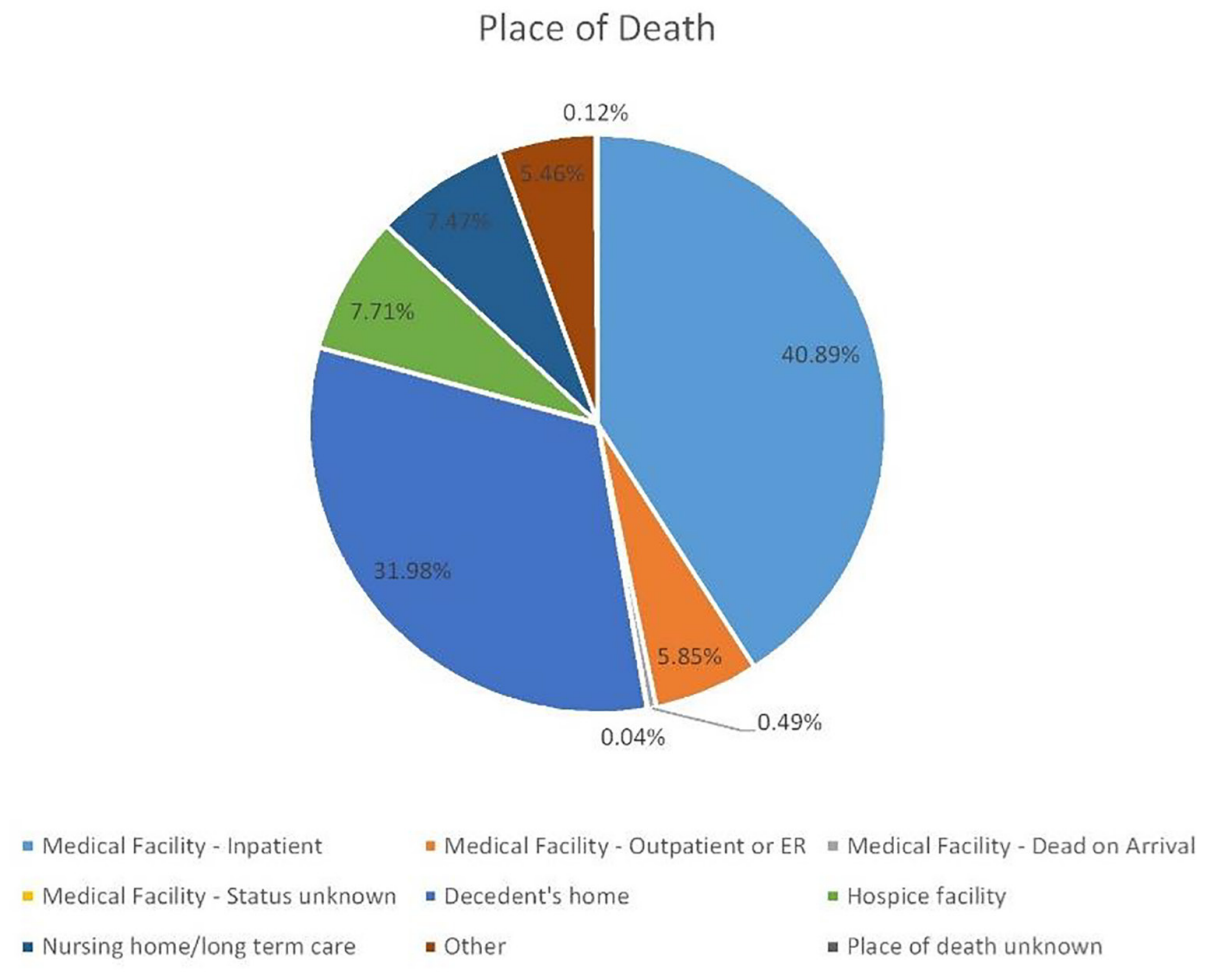
Distribution of deaths (n = 80,403) attributable to comorbid viral hepatitis C and psychoactive substance use disorders by place of death, United States, 1999–2023.

Between 1999 and 2023, a total of 80,403 deaths from comorbid viral hepatitis C and psychoactive SUD were recorded in the United States, with 71.2% among males (57,268 deaths) and 23.7% among females (19,068 deaths) (Supplemental Table 1, Supplemental Digital Content 1). By race/ethnicity, NH Whites contributed 66.6% (53,537 deaths), followed by NH Blacks 16.3% (13,071 deaths), Hispanics 10.2% (8234 deaths), NH American Indians/Alaska Natives 1.8% (1454 deaths), and NH Asians/Pacific Islanders 0.6% (479 deaths) (Supplemental Table 1, Supplemental Digital Content 1). Most deaths occurred in those aged 55–74 years (55.5%, 44,625 deaths) and 35–54 years (33.3%, 26,760 deaths), with smaller proportions among those aged ≥ 75 years (2.8%, 2230 deaths) and 15–34 years (1.8%, 1482 deaths) (Supplemental Table 2, Supplemental Digital Content 2). Regionally, the South accounted for 37.0% (29,783 deaths), followed by the West 28.5% (22,883 deaths), the Northeast 17.4% (13,996 deaths), and the Midwest 14.8% (11,891 deaths) (Supplemental Table 2, Supplemental Digital Content 2). Urban areas contributed 82.6% (63,013 deaths) compared with 17.4% in rural areas (13,323 deaths) (Supplemental Table 3, Supplemental Digital Content 3). Place of death analysis showed that 47.3% of deaths occurred in medical facilities (43,081 deaths), primarily inpatient (36.2%), while 52.7% occurred outside medical settings (33,253 deaths), including at home (31.9%), in hospice (7.7%), and in nursing homes or long-term care (7.5%) (Fig. 1) (Supplemental Table 4, Supplemental Digital Content 4).

### 3.2. Annual percent change (APC)

**Figure 2.**
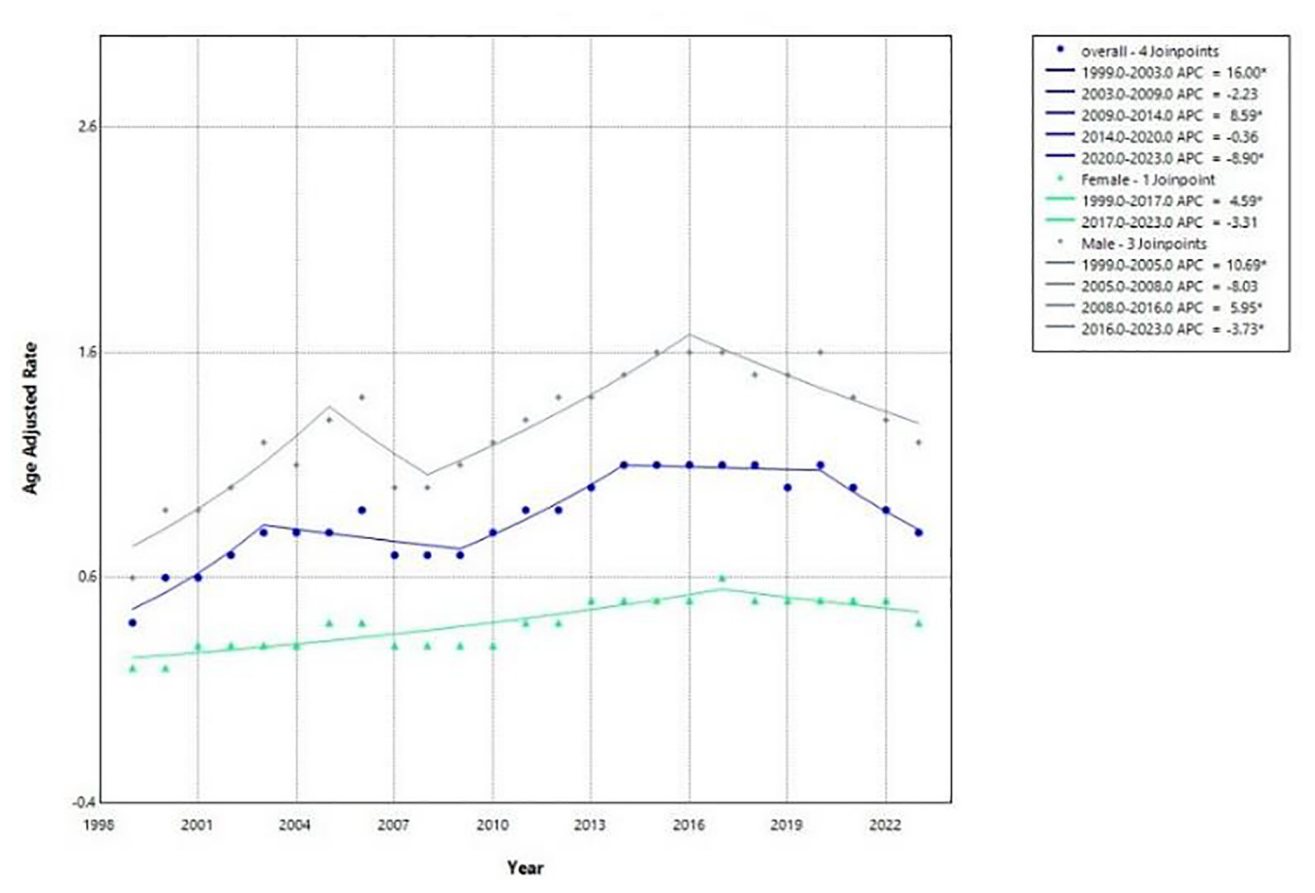
Joinpoint model of viral hepatitis C and psychoactive substance use disorders, age-adjusted mortality rates per 100,000 people, 1999–2023, Overall and stratified by sex.

Between 1999 and 2023, mortality from comorbid viral hepatitis C and psychoactive SUD demonstrated distinct temporal patterns across population subgroups. Overall mortality rose sharply between 1999 and 2003 (APC: +16.0; 95% CI: 9.7–30.3), declined modestly from 2003 to 2009 (–2.2; 95% CI:–8.2–0.3), increased again between 2009 and 2014 (+8.6; 95% CI: 5.4–14.8), and fell steeply from 2020 to 2023 (–8.9; 95% CI:–15.7 to –4.3). Males showed early increases (1999–2005: +10.7) and declines after 2016 (–3.7), while females declined initially (1999–2004:–8.9) but rose consistently thereafter (+4.6).(Fig. 2)

**Figure 3.**
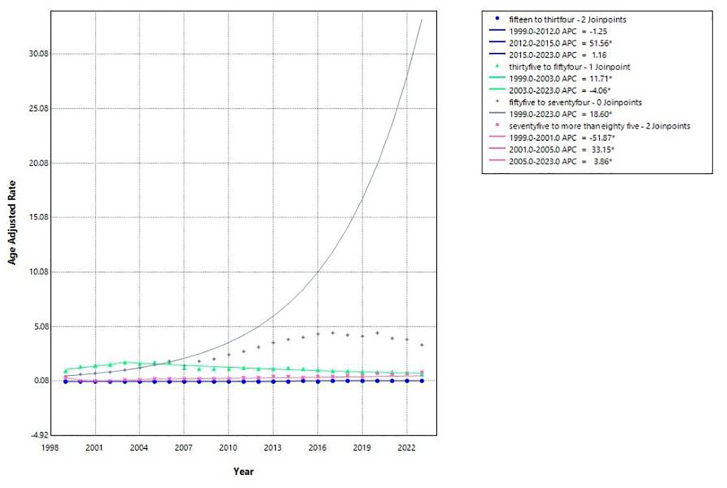
Joinpoint model of viral Hepatitis C and psychoactive substance use disorders age, age-adjusted mortality rates per 100,000 people, 1999–2023, stratified by age.

By age, deaths surged among those 55–74 years (APC: +18.6), whereas younger adults 35–54 years shifted from early increases (1999–2003: +11.7) to long-term declines (–4.1). Mortality among those ≥75 years fell initially (–51.9, 1999–2001), then rose moderately (+3.9 after 2005), while 15–34 years experienced a short-lived spike (2012–2015: +51.6). (Fig. 3)

**Figure 4.**
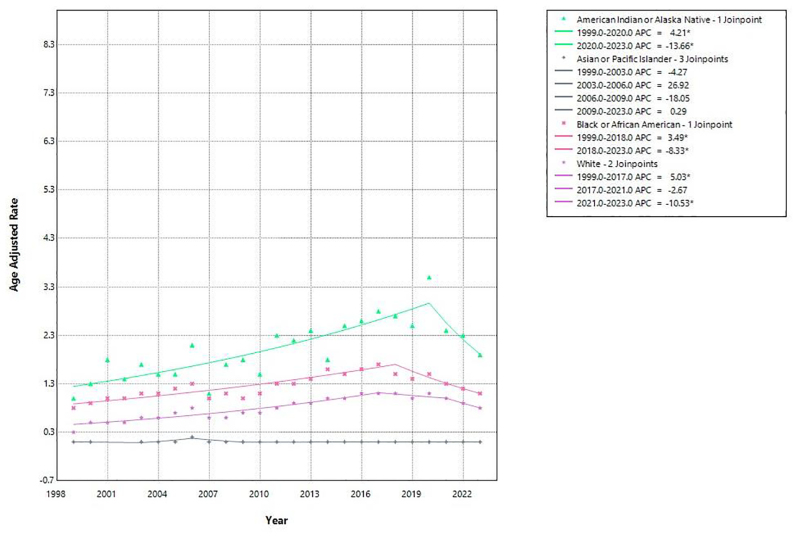
Joinpoint model of viral Hepatitis C and psychoactive substance use disorders, age-adjusted mortality rates per 100,000 people, 1999–2023, stratified by race/ethnicity.

Racial disparities were evident: NH Whites increased until 2017 (+5.0) then declined sharply after 2021 (–10.5); NH Blacks rose until 2018 (+3.5) then dropped rapidly (–8.3); NH American Indians/Alaska Natives increased until 2020 (+4.2) before a steep fall (–13.7); Hispanics rose until 2014 (+3.0) then declined (–5.3); and NH Asians/Pacific Islanders showed largely stable trends (Fig. 4)

**Figure 5.**
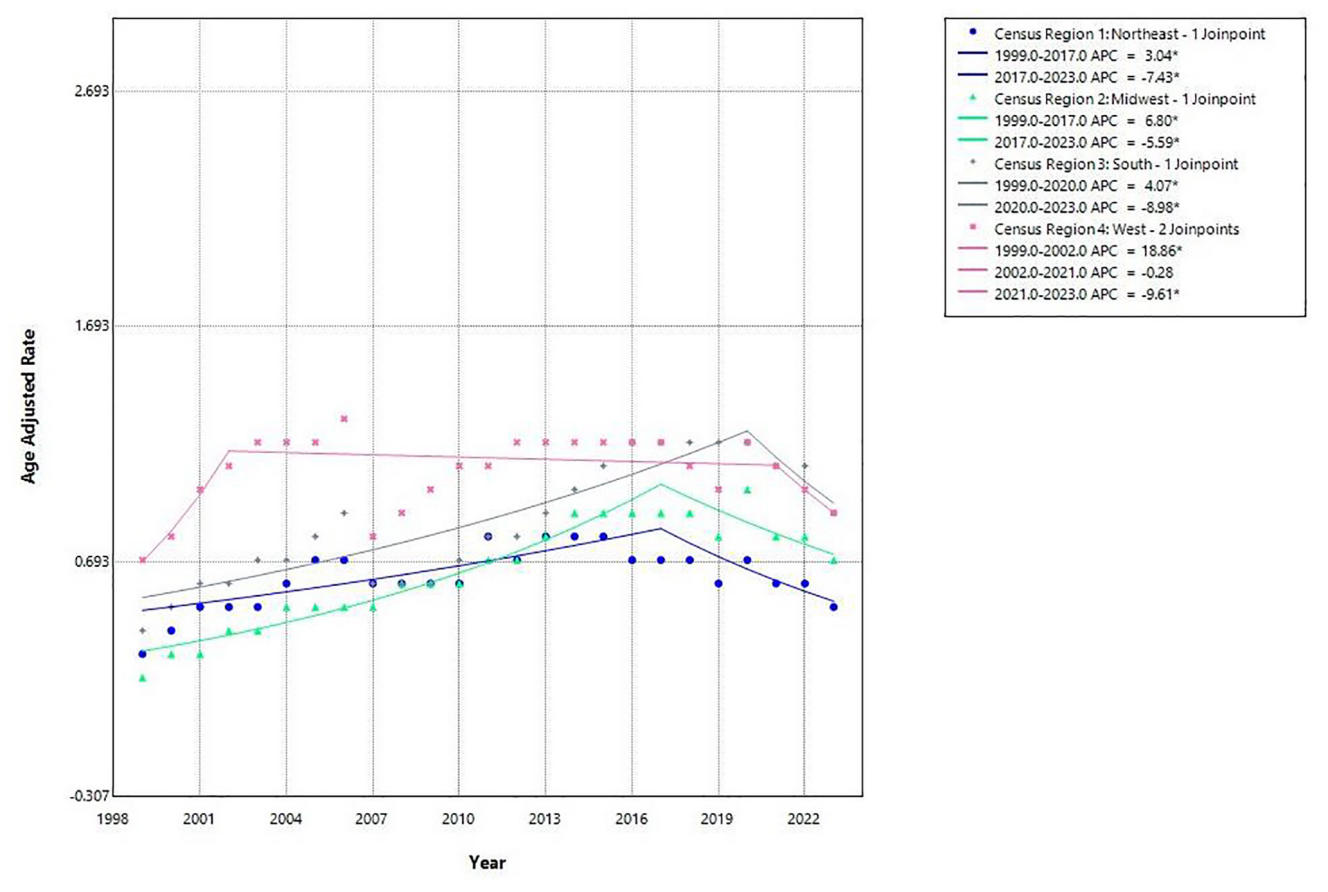
Joinpoint model of viral hepatitis C and psychoactive substance use disorders, Age-Adjusted Mortality Rates per 100,000 people, 1999–2023, stratified by region.

**Figure 6.**
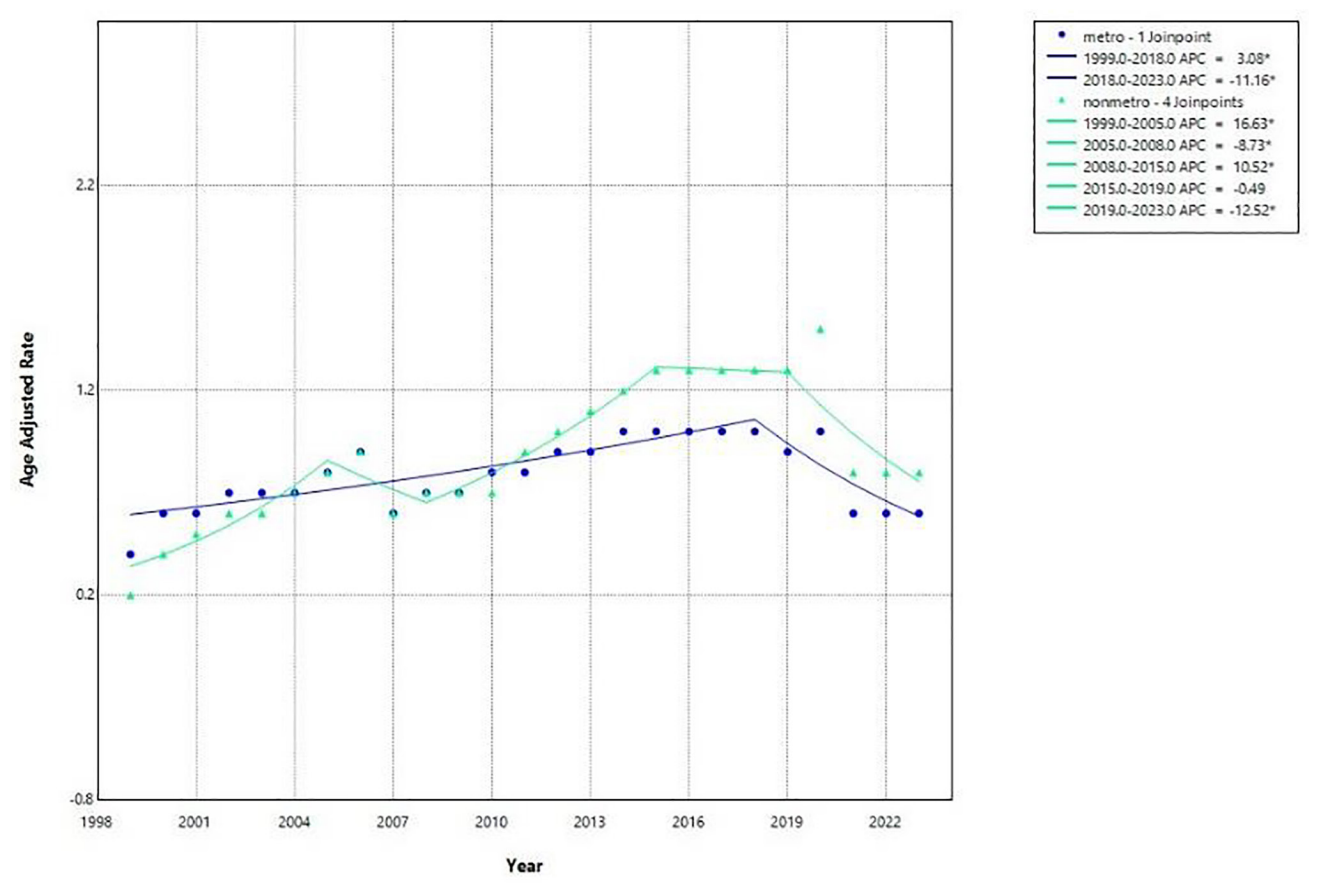
Joinpoint model of viral hepatitis C and psychoactive substance use disorders age-adjusted mortality rates per 100,000 people, 1999–2023, stratified by urban/rural status.

Regionally, the Midwest (+6.8 until 2017) and South (+4.1 until 2020) had strong growth followed by declines (–5.6 and–9.0, respectively), while the West rose early (+18.9, 1999–2002) then declined after 2021 (–9.6), and the Northeast showed modest early gains (+3.0) before later declines (–7.4) (Fig. 5). Urban mortality rose steadily until 2018 (+3.1) before falling (–11.2), while rural areas saw sharper fluctuations with early growth (+16.6, 1999–2005), a mid-period rise (+10.5, 2008–2015), and steep late declines (–12.5, 2019–2023). (Fig. 6) (Supplemental Table 5, Supplemental Digital Content 5)

### 3.3. Overall and sex stratified AAMR

**Figure 7.**
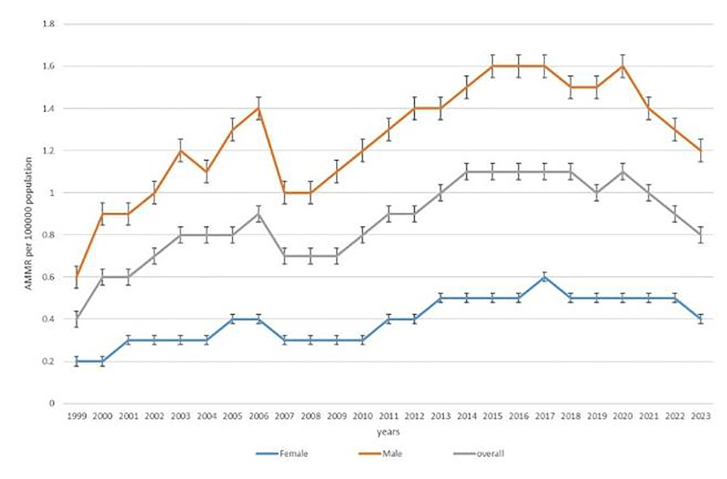
Viral hepatitis C and psychoactive substance use disorders age-adjusted mortality rate per 100,000 people, 1999–2023. Overall and stratified by sex.

The AAMR for deaths from comorbid viral hepatitis C and psychoactive SUD was 0.4 per 100,000 in 1999 and peaked at 1.1 during 2014–2020, before declining to 0.8 in 2023 (Supplemental Table 6, Supplemental Digital Content 6). Overall, the mortality burden rose steadily in the early years, plateaued during the mid-2010s, and showed a downward trend in the last 3 years of the study period. Males had consistently higher AAMRs than females throughout the study period, with overall AAMRs of 1.2 (95% CI: 1.3–1.2) for men compared with 0.3 (95% CI: 0.4–0.3) for women (Supplemental Table 6, Supplemental Digital Content 6). In 1999, male AAMR was 0.6, which increased to 1.6 in 2016 and then declined to 1.2 by 2023. Female AAMR rose more gradually, starting at 0.2 in 1999, reaching 0.6 in 2017, and ending at 0.4 in 2023. (Fig. 7)

### 3.4. AAMR stratified by age group

**Figure 8.**
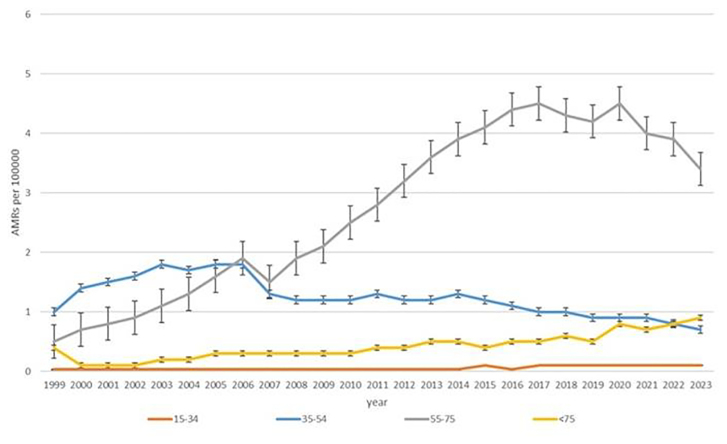
Viral hepatitis C and psychoactive substance use disorders age-adjusted mortality rate per 10,000 per 100,000 people, 1999–2023. Stratified by age.

When stratified by age, the steepest rise occurred among individuals aged 55–74 years, with AAMR increasing from 0.5 in 1999 to 4.5 in 2020, followed by a decline to 3.4 in 2023 (Supplemental Table 7, Supplemental Digital Content 7). The 35–54year group had intermediate levels, peaking around 1.8 in the early 2000s and stabilizing near 0.7–0.9 by the end of the study. Younger adults aged 15–34 years maintained very low rates (~0.03–0.1 across all years), while those ≥75 years showed a modest but steady rise from 0.1 in 2000 to 0.9 in 2023. (Fig. 8)

### 3.5. AAMR stratified by race/ethnicity

**Figure 9.**
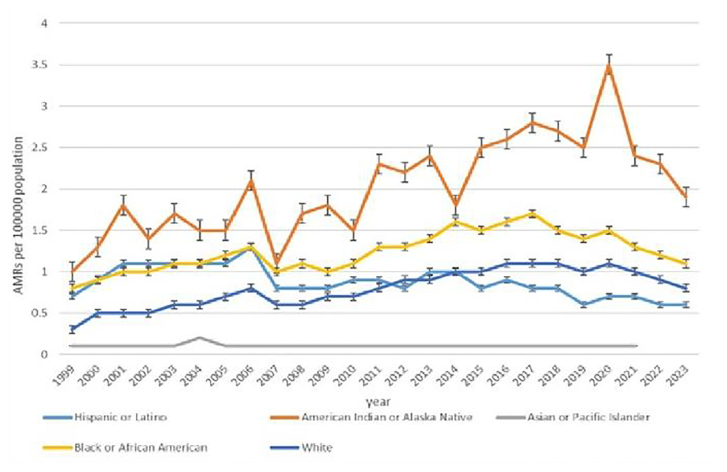
Viral hepatitis C and psychoactive substance use disorders age-adjusted mortality rate per 100,000 people, 1999–2023. Stratified by race and ethnicity.

Clear racial disparities were observed in AAMRs (Supplemental Table 8, Supplemental Digital Content 8). NH American Indians or Alaska Natives had the highest overall rate (2.0; 95% CI: 1.5–2.6), followed by NH Blacks (1.2; 95% CI: 1.1–1.3), Hispanics (0.8; 95% CI: 0.7–0.9), NH Whites (0.7; 95% CI: 0.7–0.8), and NH Asians/Pacific Islanders (0.1; 95% CI: 0.08–0.1). AAMRs among NH Whites rose steadily until 2014 (1.0) before declining slightly to 0.8 in 2023, while NH Blacks peaked at 1.7 in 2017, then fell to 1.1 in 2023. NH American Indians/Alaska Natives showed persistently high rates throughout, peaking at 2.8 in 2017. (Fig. 9)

### 3.6. AAMR stratified by geographic region

**Figure 10.**
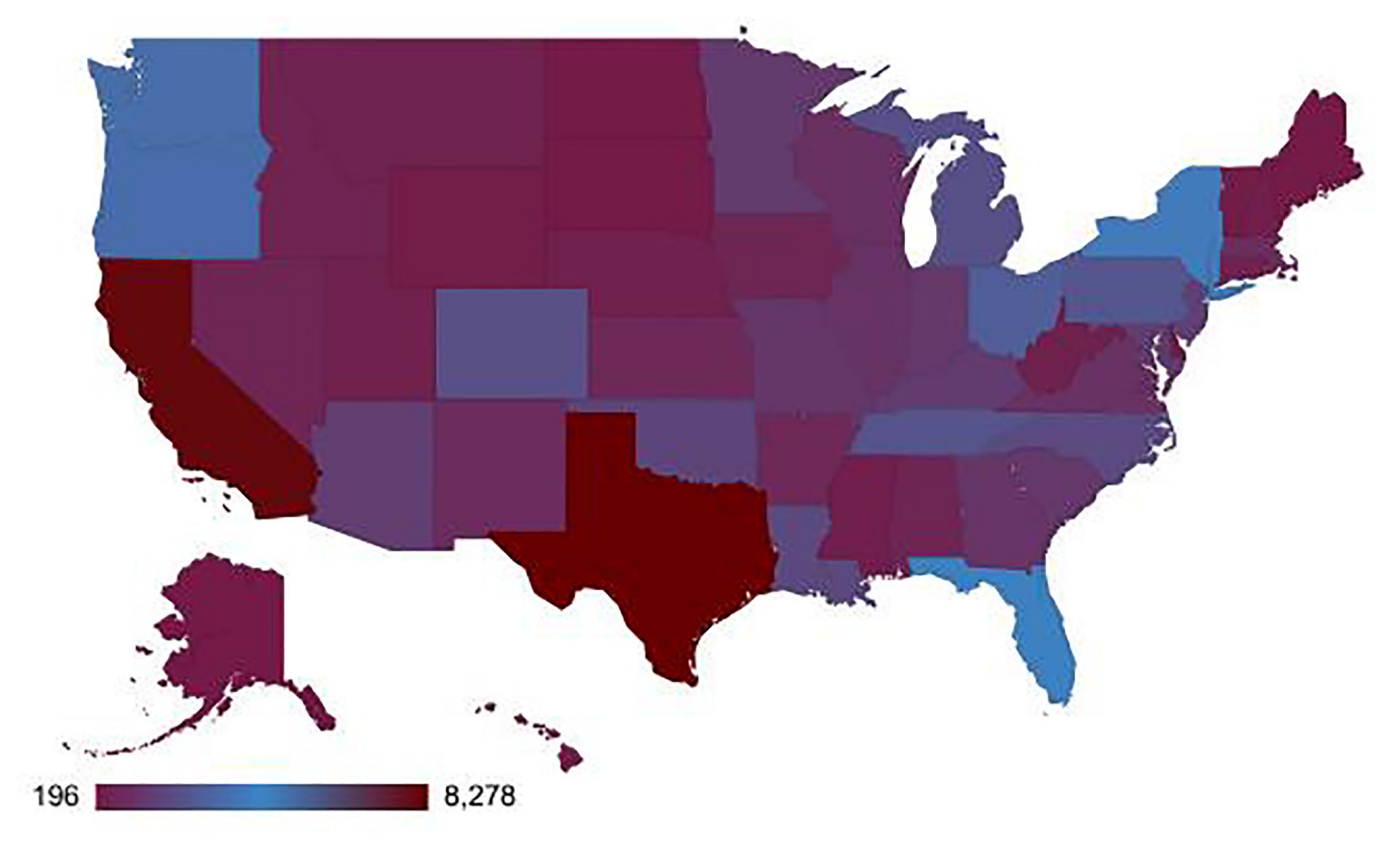
Geographical distribution of age-adjusted mortality rate per 100,000 people across states in the USA from 1999–2023.

When examined at the state level, Colorado, Louisiana, Montana, Oklahoma, the District of Columbia, and Oregon fell into the top 10th percentile of AAMRs, highlighting states with the highest mortality burden compared with the national distribution (Supplemental Table 9, Supplemental Digital Content 9). (Fig. 10)

### 3.7. AAMR stratified by geographic region

**Figure 11.**
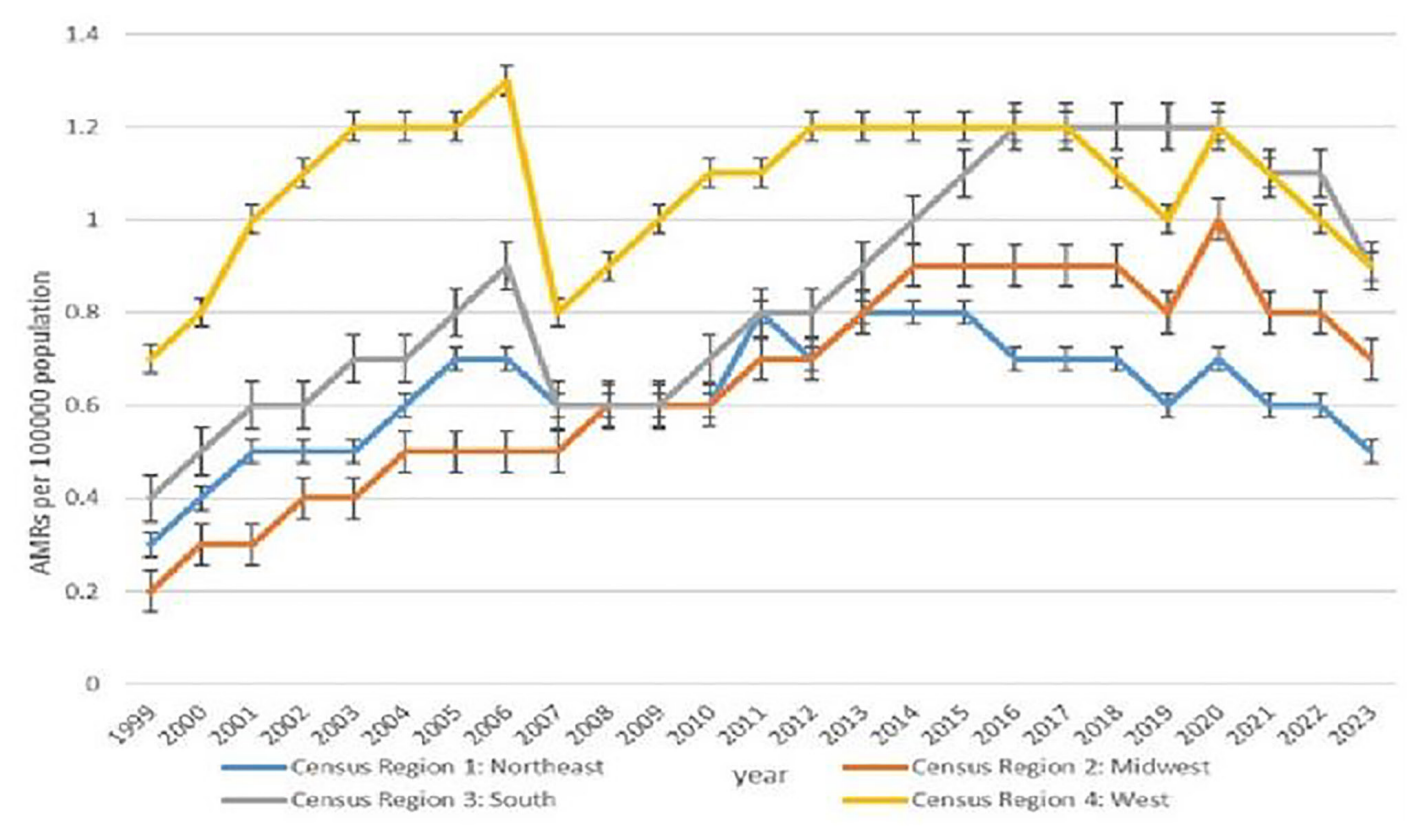
Viral hepatitis C and psychoactive substance use disorders age-adjusted mortality rate per 100,000 people, 1999–2023. Stratified by region.

Regional patterns revealed the highest AAMRs in the West (overall 1.08; 95% CI: 1.0–1.1), followed by the South (0.86; 95% CI: 0.8–0.9), Midwest (0.65; 95% CI: 0.6–0.7), and Northeast (0.62; 95% CI: 0.5–0.7) (Supplemental Table 10, Supplemental Digital Content 10). Rates in the West peaked during the early 2000s (1.3 in 2002) and remained elevated compared with other regions throughout the study period, while the Northeast consistently had the lowest levels. (Fig. 11)

### 3.8. AAMR stratified by urban–rural status

**Figure 12.**
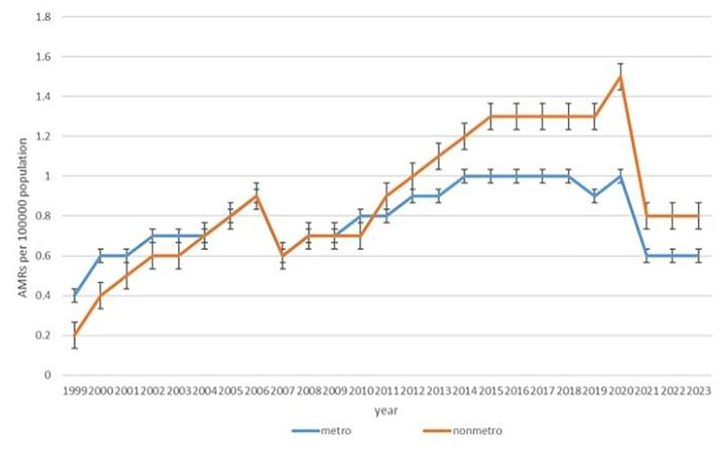
Viral hepatitis C and psychoactive substance use disorders age-adjusted mortality rate per 100,000 people, 1999–2023. Stratified by urban-rural status.

Rural populations experienced higher mortality than urban populations, with overall AAMRs of 0.89 (95% CI: 0.87–0.91) compared with 0.80 (95% CI: 0.79–0.82) (Supplemental Table 11, Supplemental Digital Content 11). Both urban and rural areas showed rising rates through the mid-2010s, peaking at 1.0 and 1.5, respectively, in 2020, followed by declines by 2023. (Fig. 12)

## 4. Discussion

In this analysis of national mortality data from the Centers for Disease Control and Prevention, we report several key findings. First, mortality from comorbid viral hepatitis C and psychoactive SUD increased steadily from 1999, peaked during 2014–2020, and then declined modestly by 2023. Second, men consistently exhibited higher mortality rates compared with women, and individuals aged 55–74 years carried the greatest burden, although notable increases were also observed in younger adults. Third, NH American Indian/Alaska Native and NH Black populations experienced disproportionately higher mortality, whereas NH Asian populations had the lowest rates. Fourth, significant geographic heterogeneity was evident, with several states, including Oregon, Oklahoma, Colorado, Louisiana, Montana, and the District of Columbia, falling into the highest decile of mortality, and rural areas experiencing consistently higher AAMRs compared with urban counterparts. These results highlight the syndemic impact of substance use and HCV across multiple demographic and structural domains (Central Illustration).

According to our findings, which are consistent with previous studies, the increased mortality burden in the early years can be partly ascribed to the rise in injection drug use nationwide^[9]^ and the overlapping opioid epidemic in the United States, which started in the mid-1990s with an increase in prescription opioid use brought on by aggressive marketing and changes in pain management. Between 1999 and 2021, the number of overdose deaths increased by almost 6 times, as the drug progressed from prescription opioids to heroin injection and finally to powerful synthetic opioids like fentanyl.^[12–15]^ The stabilization of HCV-related mortality during the mid-2010s parallels the introduction of direct-acting antivirals, which offered highly effective cure rates; however, early access was limited by high costs and restrictive state Medicaid policies that often confined treatment to patients with advanced fibrosis or cirrhosis, contrary to AASLD/IDSA recommendations.^[16,17]^ The subsequent decline in mortality after 2020 may reflect the WHO released interim guidance in 2021 for country validation of viral hepatitis elimination, with the inclusion of new absolute targets (annual incidence ≤5 per 100 000 and annual mortality ≤2 per 100 000),^[18]^ COVID-19 pandemic also significantly disrupts the delivery of healthcare, which may have contributed to observed decreases in part due to temporary underreporting or delayed care.^[19]^

Men continuously had greater death rates than women in our sample, which is consistent with other research on hepatocellular carcinoma related mortality.^[20]^ The observed racial and regional disparities underscore the deep structural inequities shaping health outcomes, consistent with previous studies showing that the national HCV-related mortality rate among American Indian/Alaska Native (AI/AN) persons was more than 2.5 times that of non-Hispanic White persons in 2016,^[21]^ due to historical trauma, limited healthcare infrastructure, and socioeconomic disadvantage. In a similar vein, systematic racism, limited access to specialist care, and greater imprisonment rates may be responsible for the higher mortality among Black communities. These factors increase the risk of HCV transmission and hinder treatment continuity.^[22]^ Multiple studies across different healthcare delivery systems have shown that Black individuals have lower odds of treatment initiation, with a ORs ranging from 0.59 to 0.93.^[23–25]^ Rural populations remain uniquely vulnerable because of healthcare workforce shortages, hospital closures, and limited access to harm reduction services such as syringe exchange programs, possibly driven by decreased access to HCV screening or availability of highly effective direct-acting antiviral therapies for rural residents.^[13,26,27]^ Collectively, these findings reinforce the need for tailored interventions that extend beyond antiviral therapy to address social determinants of health.^[28]^

The importance of these temporal and demographic shifts cannot be overstated. The rising mortality among younger adults represents a shifting epidemiologic pattern with profound implications for public health planning, social stability, and long-term economic burden, emphasizing the urgency of targeted prevention and treatment efforts.^[8,9,12]^ The persistence of high mortality in rural and marginalized communities indicates that national elimination goals will remain unattainable without addressing systemic inequities. The decline observed in the past few years offers cautious optimism but also signals an opportunity to reinforce, rather than retreat from, investments in prevention, treatment, and harm reduction.

This study has limitations. The use of death certificate data may underestimate true mortality, as misclassification and underreporting of HCV and SUD are well documented. ICD coding practices can change over time, influencing trend interpretation. The ecological design precludes causal inference at the individual level, and residual confounding by comorbidities such as HIV or alcohol-related liver disease cannot be excluded. Finally, lack of granular data on treatment uptake, socioeconomic status, and behavioral risk factors limits our ability to fully contextualize observed disparities.

Despite these limitations, our study provides a comprehensive national assessment of comorbid HCV and SUD mortality across 3 decades, revealing critical demographic and geographic disparities. These results emphasize the urgency of integrated approaches that combine curative HCV therapy with accessible, culturally responsive substance use treatment and harm-reduction services. Addressing these intertwined epidemics is essential to reduce preventable deaths and to move meaningfully toward national hepatitis elimination and harm reduction targets.

## 5. Study limitations

There are several limitations that need to be considered. First, because of the reliance on International Statistical Classification of Diseases and Health Related Problems – 10th revision codes and death certificates, there is a risk of misclassification or omission of HCV and psychoactive SUD as causes of death. Second, the database does not include clinical variables that may better characterize disease severity, such as HCV genotype, liver function tests, or details of direct-acting antiviral therapy. Third, information on behavioral, psychiatric, and socioeconomic factors, including homelessness, incarceration, and access to harm reduction services, was not available. Last, variation in reporting practices across states and over time may affect geographic comparisons.

## 6. Conclusions

After an initial rise from 1999 to the mid-2000s, mortality from comorbid HCV and psychoactive SUD demonstrated fluctuating trends with a plateau in the mid-2010s and a decline after 2020. Highest AAMR were observed among males, NH American Indian/Alaska Native and NH Black populations, middle-aged and older adults, and residents of rural areas and the Western and Southern regions of the United States. Further efforts are needed to integrate HCV treatment with addiction services and address systemic barriers to care in order to reduce preventable mortality in this vulnerable population.

## Author contributions

**Project administration:** Masira Fatima, Muddassir Khalid.

**Supervision:** Masira Fatima, Muddassir Khalid.

**Conceptualization:** Meraal Faisal, Maliha Iqbal.

**Data curation:** Meraal Faisal, Maliha Iqbal.

**Resources:** Warda Fatima Shafee, Nawaal Furqan.

**Validation:** Warda Fatima Shafee, Rida Noor.

**Methodology:** Mahveen Iqbal.

**Software:** Nawaal Furqan.

**Writing – review & editing:** Masira Fatima, Warda Fatima Shafee, Muddassir Khalid, Umaima Ali Usmani.

**Writing – original draft:** Mahveen Iqbal, Sumia Fatima.






















