# Supplementary material for: Mortality trends of comorbid viral hepatitis C and psychoactive substance use disorders in the United States: Insights from CDC WONDER, 1999–2023
Source: Medicine (Baltimore). 2026 Jun 26;105(26):e49421. doi: 10.1097/MD.0000000000049421 (PMC13313786; doi:10.1097/MD.0000000000049421)
Supplement: Supplementary file 1 [file medi-105-e49421-s001.docx]

Supplemental Table 1: Mortality trends of comorbid Viral Hepatitis C and Psychoactive Substance Use Disorders in the United States: Insights from CDC WONDER 1999–2023.

| Year | Overall | Female | Male | NH White | NH Black or African American | NH Asian or Pacific Islander | NH American Indian or Alaska Native | Hispanic or Latino |
| --- | --- | --- | --- | --- | --- | --- | --- | --- |
| 1999 | 1210 | 238 | 813 | 608 | 240 | 10 | 21 | 163 |
| 2000 | 1662 | 297 | 1198 | 964 | 258 | 17 | 26 | 216 |
| 2001 | 1835 | 383 | 1305 | 1040 | 307 |  | 39 | 285 |
| 2002 | 2009 | 404 | 1452 | 1213 | 313 |  | 32 | 277 |
| 2003 | 2297 | 464 | 1681 | 1395 | 369 | 11 | 38 | 319 |
| 2004 | 2323 | 503 | 1675 | 1434 | 370 | 15 | 34 | 313 |
| 2005 | 2686 | 600 | 1938 | 1717 | 412 | 16 | 36 | 342 |
| 2006 | 2968 | 639 | 2167 | 1848 | 465 | 23 | 51 | 403 |
| 2007 | 2176 | 518 | 1560 | 1400 | 361 | 19 | 27 | 261 |
| 2008 | 2342 | 532 | 1703 | 1501 | 414 | 13 | 44 | 259 |
| 2009 | 2578 | 609 | 1844 | 1703 | 389 | 16 | 46 | 292 |
| 2010 | 2865 | 637 | 2074 | 1840 | 462 | 20 | 39 | 332 |
| 2011 | 3263 | 780 | 2332 | 2113 | 560 | 21 | 60 | 344 |
| 2012 | 3523 | 814 | 2517 | 2333 | 545 | 23 | 59 | 335 |
| 2013 | 3814 | 916 | 2695 | 2515 | 599 | 29 | 61 | 384 |
| 2014 | 4110 | 972 | 2936 | 2667 | 694 | 24 | 52 | 437 |
| 2015 | 4330 | 1046 | 3071 | 2921 | 691 | 28 | 74 | 376 |
| 2016 | 4482 | 1098 | 3169 | 2999 | 728 | 21 | 78 | 402 |
| 2017 | 4601 | 1145 | 3207 | 3002 | 811 | 27 | 81 | 395 |
| 2018 | 4539 | 1142 | 3109 | 3023 | 721 | 16 | 80 | 379 |
| 2019 | 4399 | 1012 | 3057 | 2893 | 719 | 28 | 76 | 329 |
| 2020 | 4790 | 1170 | 3313 | 3207 | 732 | 28 | 105 | 370 |
| 2021 | 4162 | 1159 | 3003 | 3296 | 692 | 21 | 106 | 376 |
| 2022 | 3918 | 1064 | 2854 | 3113 | 635 | 21 | 103 | 329 |
| 2023 | 3521 | 926 | 2595 | 2792 | 584 | 32 | 86 | 316 |
| Total | 80403 | 19068 | 57268 | 53537 | 13071 | 479 | 1454 | 8234 |
