# Supplementary material for: Mortality trends of comorbid viral hepatitis C and psychoactive substance use disorders in the United States: Insights from CDC WONDER, 1999–2023
Source: Medicine (Baltimore). 2026 Jun 26;105(26):e49421. doi: 10.1097/MD.0000000000049421 (PMC13313786; doi:10.1097/MD.0000000000049421)
Supplement: Supplementary file 2 [file medi-105-e49421-s002.docx]

# Supplemental Table 2: Mortality trends of comorbid Viral Hepatitis C and Psychoactive Substance Use Disorders in the United States, Stratified by Age groups and Census region , 1999 to 2023

| Year | 15-34 years | 35-54 years | 55-74 years | <75  years | Northeast | Midwest | South | West |
| --- | --- | --- | --- | --- | --- | --- | --- | --- |
| 1999 | 32 | 805 | 198 | 16 | 119 | 171 | 369 | 392 |
| 2000 | 37 | 1150 | 284 | 24 | 199 | 232 | 555 | 509 |
| 2001 | 29 | 1308 | 331 | 20 | 224 | 259 | 560 | 645 |
| 2002 | 43 | 1376 | 409 | 27 | 239 | 283 | 644 | 690 |
| 2003 | 41 | 1548 | 521 | 34 | 285 | 306 | 778 | 776 |
| 2004 | 40 | 1485 | 616 | 37 | 313 | 330 | 715 | 820 |
| 2005 | 30 | 1666 | 795 | 46 | 342 | 391 | 967 | 838 |
| 2006 | 37 | 1696 | 1025 | 48 | 363 | 459 | 1023 | 961 |
| 2007 | 30 | 1141 | 857 | 50 | 357 | 384 | 700 | 637 |
| 2008 | 26 | 1084 | 1066 | 59 | 430 | 406 | 747 | 652 |
| 2009 | 27 | 1129 | 1245 | 52 | 443 | 403 | 833 | 774 |
| 2010 | 26 | 1117 | 1503 | 65 | 496 | 425 | 922 | 868 |
| 2011 | 37 | 1213 | 1784 | 78 | 610 | 510 | 1079 | 913 |
| 2012 | 50 | 1118 | 2077 | 86 | 580 | 518 | 1202 | 1031 |
| 2013 | 40 | 1059 | 2413 | 99 | 699 | 531 | 1341 | 1040 |
| 2014 | 52 | 1152 | 2611 | 93 | 774 | 596 | 1477 | 1061 |
| 2015 | 85 | 1082 | 2854 | 96 | 774 | 577 | 1682 | 1084 |
| 2016 | 71 | 973 | 3114 | 109 | 820 | 533 | 1784 | 1130 |
| 2017 | 89 | 933 | 3213 | 117 | 818 | 537 | 1889 | 1108 |
| 2018 | 110 | 854 | 3165 | 122 | 787 | 499 | 1857 | 1108 |
| 2019 | 86 | 765 | 3107 | 109 | 751 | 467 | 1840 | 1011 |
| 2020 | 124 | 793 | 3385 | 181 | 872 | 489 | 1974 | 1148 |
| 2021 | 98 | 784 | 3120 | 160 | 775 | 440 | 1852 | 1095 |
| 2022 | 99 | 666 | 2951 | 202 | 739 | 445 | 1769 | 965 |
| 2023 | 81 | 567 | 2656 | 217 | 617 | 400 | 1578 | 926 |
| Total | 1482 | 26760 | 44625 | 2230 | 13996 | 11891 | 29783 | 22883 |
