# Supplementary material for: Mortality trends of comorbid viral hepatitis C and psychoactive substance use disorders in the United States: Insights from CDC WONDER, 1999–2023
Source: Medicine (Baltimore). 2026 Jun 26;105(26):e49421. doi: 10.1097/MD.0000000000049421 (PMC13313786; doi:10.1097/MD.0000000000049421)
Supplement: Supplementary file 3 [file medi-105-e49421-s003.docx]

**Supplemental Table 3: Mortality trends of comorbid Viral Hepatitis C and** Psychoactive Substance Use Disorders **in the United States, Stratified by Urbanization, 1999 to 2023**

| Year | Urban | Rural |
| --- | --- | --- |
| 1999 | 943 | 108 |
| 2000 | 1329 | 166 |
| 2001 | 1476 | 212 |
| 2002 | 1590 | 266 |
| 2003 | 1850 | 295 |
| 2004 | 1852 | 326 |
| 2005 | 2163 | 375 |
| 2006 | 2366 | 440 |
| 2007 | 1741 | 337 |
| 2008 | 1871 | 364 |
| 2009 | 2036 | 417 |
| 2010 | 2301 | 410 |
| 2011 | 2596 | 516 |
| 2012 | 2740 | 591 |
| 2013 | 2956 | 655 |
| 2014 | 3227 | 681 |
| 2015 | 3339 | 778 |
| 2016 | 3477 | 790 |
| 2017 | 3572 | 780 |
| 2018 | 3439 | 812 |
| 2019 | 3289 | 780 |
| 2020 | 3573 | 910 |
| 2021 | 3340 | 822 |
| 2022 | 3148 | 770 |
| 2023 | 2799 | 722 |
| Overall | 63013 | 13323 |
