# Supplementary material for: Mortality trends of comorbid viral hepatitis C and psychoactive substance use disorders in the United States: Insights from CDC WONDER, 1999–2023
Source: Medicine (Baltimore). 2026 Jun 26;105(26):e49421. doi: 10.1097/MD.0000000000049421 (PMC13313786; doi:10.1097/MD.0000000000049421)
Supplement: Supplementary file 4 [file medi-105-e49421-s004.docx]

# Supplemental Table 4: Mortality trends of comorbid Viral Hepatitis C and Psychoactive Substance Use Disorders in the United States, Stratified by Place of Death, 1999 to 2023

| Place of Death | Deaths (1999-2020) | Deaths (2021-2023) | Total | Percentage |
| --- | --- | --- | --- | --- |
| Medical Facility - Inpatient | 27623 | 3587 |  |  |
| Medical Facility - Outpatient or ER | 3780 | 685 |  |  |
| Medical Facility - Dead on Arrival | 346 | 29 |  |  |
| Medical Facility - Status unknown | 31 | N/A |  |  |
| Total (Medical Facility) | 31780 | 4301 | 36081 | 47.26% |
| Decedent's home | 19881 | 4528 |  |  |
| Hospice facility | 4670 | 1216 |  |  |
| Nursing home/long term care | 4886 | 813 |  |  |
| Other | 3424 | 741 |  |  |
| Place of death unknown | 94 | N/A |  |  |
|  | 32955 | 7298 | 40253 | 52.73% |

N/A= not available (unreliable or suppressed)
