# Supplementary material for: Mortality trends of comorbid viral hepatitis C and psychoactive substance use disorders in the United States: Insights from CDC WONDER, 1999–2023
Source: Medicine (Baltimore). 2026 Jun 26;105(26):e49421. doi: 10.1097/MD.0000000000049421 (PMC13313786; doi:10.1097/MD.0000000000049421)
Supplement: Supplementary file 5 [file medi-105-e49421-s005.docx]

# Supplemental Table 5: Annual percent change (APC) of comorbid Viral Hepatitis C and Psychoactive Substance Use Disorders in the United States, 1999 to 2023

| Year Interval | APC (95% CI) | P value |
| --- | --- | --- |
| Overall | | |
| 1999-2003 | 16.0047 (9.6784 – 30.2956) | 0.0004 |
| 2003-2009 | -2.2308 (-8.1631 – 0.3113) | 0.075585 |
| 2009-2014 | 8.5936 (5.4265 – 14.758) | 0.015197 |
| 2014-2020 | -0.357 (-2.6793 – 3.4924) | 0.776645 |
| 2020-2023 | -8.9041 (-15.7037 – -4.2962) | < 0.000001 |
| Male | | |
| 1999-2005 | 10.6863 (7.1661 19.7462) | 0.0008 |
| 2005-2008 | -8.0309 (-12.5576 0.0875) | 0.053989 |
| 2008-2016 | 5.9458 (4.1764 13.4964) | 0.003999 |
| 2016-2023 | -3.7327 (-6.6934 -1.6235) | 0.002799 |
| Female | | |
| 1999-2004 | -8.9041* (-15.7037 -4.2962) | < 0.000001 |
| 2004-2023 | 4.5878* (3.5665 10.5495) | 0.127574 |
|  | **15-34 years** |  |
| 1999–2012 | -1.25 (-12.32 to 3.74) | Not significant |
| 2012–2015 | 51.56 (20.13 to 79.26) | <0.05 |
| 2015–2023 | 1.16 (-3.06 to 3.70) | Not significant |
|  | **35-54 years** |  |
| 1999-2003 | 11.71 (4.97 to 24.48) | <0.05 |
| 2003-2023 | -4.06 (-4.99 to -3.44) | <0.05 |
|  | **55-74 years** |  |
| 1999-2023 | 18.60 (16.59 to 20.55) | <0.05 |
|  | **<75 years** |  |
| 1999-2001 | -51.87 (-62.40 to -36.39) | <0.05 |
| 2001-2005 | 33.15 (20.87 to 64.22) | <0.05 |
| 2005-2023 | 3.86 (1.73 to 5.58) | <0.05 |
| NH White | | |
| 1999-2017 | 5.0344* (3.8369 15.6255) | 0.023195 |
| 2017-2021 | -2.6677 (-4.6403 5.5656) | 0.478304 |
| 2021-2023 | -10.5328* (-11.7425-9.2792) | <0.000001 |
| NH Black or African American | | |
| 1999-2018 | 3.4936* (2.5857 4.4102) | <0.000001 |
| 2018-2023 | -8.3330* (-8.3643 -8.3050) | <0.000001 |
| NH American Indian or Alaska Native | | |
| 1999-2020 | 4.2148* (3.1711 6.1344) | <0.000001 |
| 2020-2023 | -13.6572* (-29.0893-2.2983) | 0.019596 |
| NH Asian or Pacific Islander | | |
| 1999-2003 | -4.2684 (-13.1162 9.5994) | 0.353529 |
| 2003-2006 | 26.9170 (-14.4411 41.9886) | 0.104379 |
| 2006-2009 | -18.0476 (-22.8593 8.6369) | 0.096781 |
| 2009-2023 | 0.2920 (-0.4888 2.0229) | 0.203559 |
| Hispanic or Latino | | |
| 1999-2014 | 3.0499* (1.3079 10.2120) | 0.001600 |
| 2014-2023 | -5.2854* (-7.2717 -3.8982) | <0.000001 |
| Rural areas | | |
| 1999-2005 | 16.6318* (12.12 26.8949) | 0.003599 |
| 2005-2008 | -8.7264* (-13.428 -0.0203) | 0.04959 |
| 2008-2015 | 10.5240* (8.712 17.6042) | 0.009598 |
| 2015-2019 | -0.4942 (-3.8918 5.0362) | 0.869426 |
| 2019-2023 | -12.5186* (-28.1935-9.2442) | <0.000001 |
| Urban areas | | |
| 1999-2018 | 3.0812* (2.1869 4.4368) | <0.000001 |
| 2018-2023 | -11.1594* (-20.9684 5.4225) | 0.0008 |
| Northeast | | |
| 1999-2017 | 3.0424* (1.0272 6.4523) | 0.004399 |
| 2017-2023 | -7.4260* (-10.8115 -3.7922) | <0.000001 |
| Midwest | | |
| 1999-2017 | 6.8037* (5.1347 9.6033) | <0.000001 |
| 2017-2023 | -5.5889* (-7.5336 -3.4525) | <0.000001 |
| South | | |
| 1999-2020 | 4.0693* (2.6179 6.6472) | <0.000001 |
| 2020-2023 | -8.9806* (-11.9764 -5.7431) | <0.000001 |
| West | | |
| 1999-2002 | 18.8572* (6.6788 52.2388) | 0.003599 |
| 2002-2021 | -0.2774 (-0.567 0.5893) | 0.537093 |
| 2021-2023 | -9.6115* (-11.1816 -7.9465) | <0.000001 |
