# Supplementary material for: Mortality trends of comorbid viral hepatitis C and psychoactive substance use disorders in the United States: Insights from CDC WONDER, 1999–2023
Source: Medicine (Baltimore). 2026 Jun 26;105(26):e49421. doi: 10.1097/MD.0000000000049421 (PMC13313786; doi:10.1097/MD.0000000000049421)
Supplement: Supplementary file 7 [file medi-105-e49421-s007.docx]

# Supplemental Table 7: Comorbid Viral Hepatitis C and Psychoactive Substance Use Disorders, Age-Adjusted Mortality Rates per 100,000, Stratified by age groups in the United States, 1999 to 2023

|  | Age-Adjusted Rate (95% CI) | | | |
| --- | --- | --- | --- | --- |
| Year | **15-34** | **35-54** | **55-74** | **75+** |
| 1999 | 0.03 (0.03–0.1) | 1 (0.9–1.1) | 0.5 (0.4–0.5) | 0.4 (0–0.1) |
| 2000 | 0.03 (0.03–0.1) | 1.4 (1.3–1.5) | 0.7 (0.6–0.8) | 0.1 (0.1–0.2) |
| 2001 | 0.03 (0.03–0.1) | 1.5 (1.4–1.6) | 0.8 (0.7–0.8) | 0.1 (0.0001–0.1) |
| 2002 | 0.03 (0.03–0.1) | 1.6 (1.5–1.7) | 0.9 (0.8–0.9) | 0.1 (0.1–0.2) |
| 2003 | 0.03 (0.03–0.1) | 1.8 (1.7–1.9) | 1.1 (1–1.2) | 0.2 (0.1–0.2) |
| 2004 | 0.03 (0.03–0.1) | 1.7 (1.6–1.7) | 1.3 (1.2–1.4) | 0.2 (0.1–0.2) |
| 2005 | 0.03 (0.03–0.1) | 1.8 (1.8–1.9) | 1.6 (1.4–1.7) | 0.3 (0.2–0.4) |
| 2006 | 0.03 (0.03–0.1) | 1.8 (1.7–1.9) | 1.9 (1.8–2) | 0.3 (0.2–0.4) |
| 2007 | 0.03 (0.03–0.1) | 1.3 (1.2–1.3) | 1.5 (1.4–1.6) | 0.3 (0.2–0.4) |
| 2008 | 0.03 (0.03–0.1) | 1.2 (1.1–1.2) | 1.9 (1.8–2) | 0.3 (0.3–0.5) |
| 2009 | 0.03 (0.03–0.1) | 1.2 (1.1–1.2) | 2.1 (2–2.2) | 0.3 (0.2–0.4) |
| 2010 | 0.03 (0.03–0.1) | 1.2 (1.1–1.2) | 2.5 (2.3–2.6) | 0.3 (0.2–0.4) |
| 2011 | 0.03 (0.03–0.1) | 1.3 (1.2–1.3) | 2.8 (2.7–2.9) | 0.4 (0.3–0.5) |
| 2012 | 0.03 (0.03–0.1) | 1.2 (1.1–1.3) | 3.2 (3–3.3) | 0.4 (0.3–0.5) |
| 2013 | 0.03 (0.03–0.1) | 1.2 (1.1–1.2) | 3.6 (3.5–3.8) | 0.5 (0.4–0.6) |
| 2014 | 0.03 (0.03–0.1) | 1.3 (1.2–1.3) | 3.9 (3.7–4) | 0.5 (0.4–0.6) |
| 2015 | 0.1 (0.1–0.1) | 1.2 (1.1–1.2) | 4.1 (4–4.3) | 0.4 (0.4–0.5) |
| 2016 | 0.03 (0.03–0.1) | 1.1 (1–1.1) | 4.4 (4.2–4.5) | 0.5 (0.4–0.6) |
| 2017 | 0.1 (0.1–0.1) | 1 (1–1.1) | 4.5 (4.3–4.6) | 0.5 (0.4–0.6) |
| 2018 | 0.1 (0.1–0.1) | 1 (0.9–1.1) | 4.3 (4.1–4.4) | 0.6 (0.5–0.7) |
| 2019 | 0.1 (0.1–0.1) | 0.9 (0.8–1) | 4.2 (4–4.3) | 0.5 (0.4–0.6) |
| 2020 | 0.1 (0.1–0.2) | 0.9 (0.8–1) | 4.5 (4.4–4.7) | 0.8 (0.7–0.9) |
| 2021 | 0.1 (0.1–0.1) | 0.9 (0.9–1) | 4 (3.9–4.2) | 0.7 (0.6–0.8) |
| 2022 | 0.1 (0.1–0.1) | 0.8 (0.7–0.8) | 3.9 (3.7–4) | 0.8 (0.7–1) |
| 2023 | 0.1 (0.1–0.1) | 0.7 (0.6–0.7) | 3.4 (3.3–3.6) | 0.9 (0.8–1) |
| Overall | 0.05 (.050-.11) | 1.24 (1.13–1.32) | 2.70 (2.53–2.83) | 0.42 (0.34–0.53) |
