# Supplementary material for: Mortality trends of comorbid viral hepatitis C and psychoactive substance use disorders in the United States: Insights from CDC WONDER, 1999–2023
Source: Medicine (Baltimore). 2026 Jun 26;105(26):e49421. doi: 10.1097/MD.0000000000049421 (PMC13313786; doi:10.1097/MD.0000000000049421)
Supplement: Supplementary file 8 [file medi-105-e49421-s008.docx]

# Supplemental Table 8: Comorbid Viral Hepatitis C and Psychoactive Substance Use Disorders, Age-Adjusted Mortality Rates per 100,000, Stratified by Race in the United States, 1999 to 2023

|  | Age-Adjusted Rate (95% CI) | | | | |
| --- | --- | --- | --- | --- | --- |
| Year | **NH White** | **NH Black or African American** | **NH American Indian or Alaska Native** | **NH Asian or Pacific Islander** | **Hispanic or Latino** |
| 1999 | 0.3  (0.3–0.3) | 0.8  (0.7–0.9) | 1  (0.6–1.6) | 0.1  (0.1–0.2) | 0.7  (0.6–0.9) |
| 2000 | 0.5  (0.4–0.5) | 0.9  (0.8–1) | 1.3  (0.8–1.9) | 0.1  (0.1–0.3) | 0.9  (0.8–1) |
| 2001 | 0.5  (0.5–0.5) | 1  (0.9–1.1) | 1.8  (1.3–2.5) | unreliable | 1.1  (1–1.2) |
| 2002 | 0.5  (0.5–0.6) | 1  (0.9–1.1) | 1.4  (1–2) | unreliable | 1.1  (0.9–1.2) |
| 2003 | 0.6  (0.6–0.7) | 1.1  (1–1.2) | 1.7  (1.2–2.3) | 0.1  (0.001–0.2) | 1.1  (1–1.3) |
| 2004 | 0.6  (0.6–0.7) | 1.1  (1–1.2) | 1.5  (1–2.1) | 0.1  (0.1–0.2) | 1.1  (1–1.2) |
| 2005 | 0.7  (0.7–0.8) | 1.2  (1.1–1.3) | 1.5  (1.1–2.1) | 0.1  (0.1–0.2) | 1.1  (1–1.2) |
| 2006 | 0.8  (0.7–0.8) | 1.3  (1.2–1.4) | 2.1  (1.6–2.7) | 0.2  (0.1–0.3) | 1.3  (1.1–1.4) |
| 2007 | 0.6  (0.5–0.6) | 1  (0.9–1.1) | 1.1  (0.7–1.6) | 0.1  (0.1–0.2) | 0.8  (0.7–0.9) |
| 2008 | 0.6  (0.6–0.6) | 1.1  (1–1.2) | 1.7  (1.3–2.3) | 0.1  (0.001–0.1) | 0.8  (0.7–0.9) |
| 2009 | 0.7  (0.6–0.7) | 1  (0.9–1.1) | 1.8  (1.3–2.4) | 0.1  (0.1–0.2) | 0.8  (0.7–0.9) |
| 2010 | 0.7  (0.7–0.7) | 1.1  (1–1.3) | 1.5  (1–2) | 0.1  (0.1–0.2) | 0.9  (0.8–1) |
| 2011 | 0.8  (0.8–0.8) | 1.3  (1.2–1.4) | 2.3  (1.7–2.9) | 0.1  (0.1–0.2) | 0.9  (0.8–1) |
| 2012 | 0.9  (0.8–0.9) | 1.3  (1.2–1.4) | 2.2  (1.6–2.8) | 0.1  (0.1–0.2) | 0.8  (0.8–0.9) |
| 2013 | 0.9  (0.9–1) | 1.4  (1.3–1.5) | 2.4  (1.8–3.1) | 0.1  (0.1–0.2) | 1  (0.9–1.1) |
| 2014 | 1  (0.9–1) | 1.6  (1.4–1.7) | 1.8  (1.4–2.4) | 0.1  (0.1–0.2) | 1  (0.9–1.1) |
| 2015 | 1  (1–1.1) | 1.5  (1.4–1.6) | 2.5  (2–3.2) | 0.1  (0.1–0.2) | 0.8  (0.8–0.9) |
| 2016 | 1.1  (1–1.1) | 1.6  (1.4–1.7) | 2.6  (2–3.3) | 0.1  (0.1–0.2) | 0.9  (0.8–0.9) |
| 2017 | 1.1  (1–1.1) | 1.7  (1.6–1.8) | 2.8  (2.2–3.5) | 0.1  (0.1–0.2) | 0.8  (0.7–0.9) |
| 2018 | 1.1  (1–1.1) | 1.5  (1.4–1.6) | 2.7  (2.1–3.4) | 0.1  (0.00-0.1) | 0.8  (0.7–0.8) |
| 2019 | 1 (1–1.1) | 1.4  (1.3–1.5) | 2.5  (2–3.2) | 0.1  (0.1–0.2) | 0.6  (0.6–0.7) |
| 2020 | 1.1  (1.1-1.2) | 1.5  (1.4–1.6) | 3.5  (2.8–4.2) | 0.1  (0.1–0.2) | 0.7  (0.6–0.8) |
| 2021 | 1  (1–1) | 1.3  (1.2–1.4) | 2.4  (1.9–2.8) | 0.1  (0–0.1) | 0.7  (0.6–0.7) |
| 2022 | 0.9  (0.9–1) | 1.2  (1.1–1.3) | 2.3  (1.9–2.8) | 0.1  (0.1–0.1) | 0.6  (0.5–0.7) |
| 2023 | 0.8  (0.80.9) | 1.1  (1–1.2) | 1.9  (1.6–2.4) | 0.1  (0.1–0.2) | 0.6  (0.5–0.6) |
| Overall | 0.7  (0.70.8) | 1.2  (1.1-1.3) | 2.0  (1.5-2.6) | 0.1  (0.08-0.1) | 0.8  (0.0.7-0.9) |
