# Supplementary material for: Mortality trends of comorbid viral hepatitis C and psychoactive substance use disorders in the United States: Insights from CDC WONDER, 1999–2023
Source: Medicine (Baltimore). 2026 Jun 26;105(26):e49421. doi: 10.1097/MD.0000000000049421 (PMC13313786; doi:10.1097/MD.0000000000049421)
Supplement: Supplementary file 9 [file medi-105-e49421-s009.docx]

# Supplemental Table 9: Comorbid Viral Hepatitis C and Psychoactive Substance Use Disorders, Age-Adjusted Mortality Rates per 100,000, Stratified by States in the United States, 1999 to 2023

| State | Age-Adjusted Rate (95%CI) | |
| --- | --- | --- |
|  | **1999-2020** | **2021-2023** |
| Alabama | 0.4 (0.3–0.4) | 0.7 (0.6–0.8) |
| Alaska | 1.1 (0.9–1.3) | 1.7 (1.2–2.3) |
| Arizona | 0.9 (0.8–0.9) | 0.8 (0.6–0.9) |
| Arkansas | 0.8 (0.8–0.9) | 1.0 (0.8–1.2) |
| California | 0.9 (0.8–0.9) | 0.5 (0.5–0.5) |
| Colorado | 1.5 (1.4–1.5) | 2.0 (1.8–2.2) |
| Connecticut | 0.5 (0.4–0.5) | 0.3 (0.2–0.4) |
| Delaware | 0.9 (0.8–1.0) | 1.0 (0.7–1.4) |
| District of Columbia | 2.3 (2.1–2.6) | 1.9 (1.3–2.5) |
| Florida | 0.8 (0.7–0.8) | 0.7 (0.6–0.8) |
| Georgia | 0.5 (0.4–0.5) | 0.4 (0.4–0.5) |
| Hawaii | 0.8 (0.7–0.9) | 0.4 (0.2–0.5) |
| Idaho | 1.2 (1.1–1.3) | 1.1 (0.8–1.3) |
| Illinois | 0.3 (0.3–0.3) | 0.4 (0.3–0.4) |
| Indiana | 0.7 (0.7–0.7) | 0.8 (0.7–0.9) |
| Iowa | 0.7 (0.7–0.8) | 0.8 (0.6–0.9) |
| Kansas | 0.9 (0.9–1.0) | 1.0 (0.8–1.2) |
| Kentucky | 1.0 (1.0–1.1) | 2.2 (1.9–2.4) |
| Louisiana | 1.4 (1.3–1.5) | 2.1 (1.9–2.3) |
| Maine | 0.5 (0.4–0.6) | 0.5 (0.4–0.8) |
| Maryland | 0.9 (0.8–0.9) | 0.9 (0.8–1.1) |
| Massachusetts | 0.5 (0.4–0.5) | 0.6 (0.5–0.7) |
| Michigan | 0.8 (0.7–0.8) | 0.7 (0.6–0.8) |
| Minnesota | 0.9 (0.8–0.9) | 1.2 (1.1–1.4) |
| Mississippi | 0.4 (0.4–0.5) | 0.8 (0.6–1.0) |
| Missouri | 0.8 (0.7–0.8) | 0.7 (0.6–0.8) |
| Montana | 1.6 (1.4–1.7) | 1.9 (1.4–2.4) |
| Nebraska | 0.9 (0.8–0.9) | 0.8 (0.6–1.0) |
| Nevada | 0.8 (0.7–0.8) | 0.8 (0.6–0.9) |
| New Hampshire | 0.8 (0.7–0.9) | 0.6 (0.4–0.9) |
| New Jersey | 0.6 (0.6–0.7) | 0.5 (0.4–0.5) |
| New Mexico | 1.7 (1.6–1.8) | 1.3 (1.1–1.6) |
| New York | 0.7 (0.7–0.7) | 0.4 (0.4–0.5) |
| North Carolina | 0.7 (0.7–0.8) | 0.8 (0.7–0.8) |
| North Dakota | 0.9 (0.8–1.1) | 2.1 (1.5–2.7) |
| Ohio | 0.8 (0.7–0.8) | 0.9 (0.8–1.0) |
| Oklahoma | 1.6 (1.5–1.7) | 2.5 (2.3–2.8) |
| Oregon | 2.8 (2.7–2.9) | 3.1 (2.8–3.4) |
| Pennsylvania | 0.6 (0.6–0.6) | 0.8 (0.7–0.9) |
| Rhode Island | 1.5 (1.3–1.6) | 1.1 (0.8–1.5) |
| South Carolina | 0.9 (0.9–1.0) | 1.0 (0.9–1.2) |
| South Dakota | 0.7 (0.6–0.9) | 1.5 (1.1–2.0) |
| Tennessee | 1.2 (1.1–1.2) | 2.0 (1.8–2.2) |
| Texas | 1.3 (1.2–1.3) | 1.0 (1.0–1.1) |
| Utah | 0.7 (0.6–0.8) | 0.4 (0.3–0.6) |
| Vermont | 1.3 (1.1–1.4) | 1.8 (1.3–2.5) |
| Virginia | 0.4 (0.4–0.4) | 0.8 (0.7–1.0) |
| Washington | 1.7 (1.6–1.8) | 1.6 (1.5–1.8) |
| West Virginia | 0.7 (0.6–0.8) | 2.6 (2.1–3.0) |
| Wisconsin | 0.6 (0.6–0.6) | 0.7 (0.6–0.8) |
| Wyoming | 1.5 (1.3–1.7) | 1.8 (1.3–2.5) |

|  |  |  |
| --- | --- | --- |
|  |  |  |

N/A = not available (unreliable or suppressed)
