# Supplementary material for: Mortality trends of comorbid viral hepatitis C and psychoactive substance use disorders in the United States: Insights from CDC WONDER, 1999–2023
Source: Medicine (Baltimore). 2026 Jun 26;105(26):e49421. doi: 10.1097/MD.0000000000049421 (PMC13313786; doi:10.1097/MD.0000000000049421)
Supplement: Supplementary file 10 [file medi-105-e49421-s010.docx]

# Supplemental Table 10: Comorbid Viral Hepatitis C and Psychoactive Substance Use Disorders, Age-Adjusted Mortality Rates per 100,000, Stratified by Census Region in the United States, 1999 to 2023

|  | Age-Adjusted Rate (95% CI) | | | |
| --- | --- | --- | --- | --- |
| Year | **Northeast** | **Midwest** | **South** | **West** |
| 1999 | 0.3 (0.3–0.4) | 0.2 (0.1–0.2) | 0.4 (0.3–0.4) | 0.7 (0.6–0.7) |
| 2000 | 0.4 (0.4–0.5) | 0.3 (0.3–0.4) | 0.5 (0.5–0.6) | 0.8 (0.8–0.9) |
| 2001 | 0.5 (0.4–0.5) | 0.3 (0.3–0.4) | 0.6 (0.5–0.6) | 1.0 (0.9–1.1) |
| 2002 | 0.5 (0.4–0.6) | 0.4 (0.3–0.4) | 0.6 (0.6–0.7) | 1.1 (1.0–1.2) |
| 2003 | 0.5 (0.5–0.6) | 0.4 (0.4–0.5) | 0.7 (0.7–0.8) | 1.2 (1.1–1.2) |
| 2004 | 0.6 (0.5–0.6) | 0.5 (0.4–0.5) | 0.7 (0.6–0.7) | 1.2 (1.1–1.3) |
| 2005 | 0.7 (0.6–0.7) | 0.5 (0.4–0.5) | 0.8 (0.8–0.9) | 1.2 (1.1–1.3) |
| 2006 | 0.7 (0.7–0.8) | 0.5 (0.4–0.5) | 0.9 (0.8–0.9) | 1.3 (1.2–1.4) |
| 2007 | 0.6 (0.6–0.7) | 0.5 (0.4–0.5) | 0.6 (0.5–0.6) | 0.8 (0.8–0.9) |
| 2008 | 0.6 (0.6–0.7) | 0.6 (0.5–0.6) | 0.6 (0.5–0.6) | 0.9 (0.8–0.9) |
| 2009 | 0.6 (0.6–0.7) | 0.6 (0.5–0.6) | 0.6 (0.6–0.7) | 1.0 (0.9–1.1) |
| 2010 | 0.6 (0.6–0.7) | 0.6 (0.6–0.7) | 0.7 (0.7–0.8) | 1.1 (1.0–1.1) |
| 2011 | 0.8 (0.7–0.8) | 0.7 (0.7–0.8) | 0.8 (0.7–0.8) | 1.1 (1.0–1.2) |
| 2012 | 0.7 (0.7–0.8) | 0.7 (0.7–0.8) | 0.8 (0.8–0.9) | 1.2 (1.2–1.3) |
| 2013 | 0.8 (0.7–0.8) | 0.8 (0.8–0.9) | 0.9 (0.9–1.0) | 1.2 (1.1–1.3) |
| 2014 | 0.8 (0.8–0.9) | 0.9 (0.8–1.0) | 1.0 (1.0–1.1) | 1.2 (1.1–1.3) |
| 2015 | 0.8 (0.7–0.9) | 0.9 (0.8–1.0) | 1.1 (1.1–1.2) | 1.2 (1.1–1.2) |
| 2016 | 0.7 (0.7–0.8) | 0.9 (0.9–1.0) | 1.2 (1.1–1.2) | 1.2 (1.1–1.3) |
| 2017 | 0.7 (0.7–0.8) | 0.9 (0.9–1.0) | 1.2 (1.2–1.3) | 1.2 (1.1–1.2) |
| 2018 | 0.7 (0.6–0.8) | 0.9 (0.8–1.0) | 1.2 (1.1–1.2) | 1.1 (1.1–1.2) |
| 2019 | 0.6 (0.6–0.7) | 0.8 (0.8–0.9) | 1.2 (1.1–1.2) | 1.0 (1.0–1.1) |
| 2020 | 0.7 (0.6–0.7) | 1.0 (0.9–1.0) | 1.2 (1.2–1.3) | 1.2 (1.1–1.2) |
| 2021 | 0.6 (0.5–0.6) | 0.8 (0.8–0.9) | 1.1 (1.1–1.2) | 1.1 (1.0–1.2) |
| 2022 | 0.6 (0.6–0.7) | 0.8 (0.8–0.9) | 1.1 (1.0–1.1) | 1.0 (0.9–1.0) |
| 2023 | 0.5 (0.5–0.6) | 0.7 (0.6–0.7) | 0.9 (0.9–1.0) | 0.9 (0.8–1.0) |
| Overall | 0.62 (0.5-0.7) | 0.65 (0.6–0.7) | 0.86 (0.8–0.9) | 1.08 (1.0–1.1) |
