# Supplementary material for: Mortality trends of comorbid viral hepatitis C and psychoactive substance use disorders in the United States: Insights from CDC WONDER, 1999–2023
Source: Medicine (Baltimore). 2026 Jun 26;105(26):e49421. doi: 10.1097/MD.0000000000049421 (PMC13313786; doi:10.1097/MD.0000000000049421)
Supplement: Supplementary file 11 [file medi-105-e49421-s011.docx]

# Supplemental Table 11: Comorbid Viral Hepatitis C and Psychoactive Substance Use Disorders, Age-Adjusted Mortality Rates per 100,000 in United States stratified by Urban-Rural Classification, 1999-2023

|  | Age-Adjusted Rate (95% CI) | |
| --- | --- | --- |
| Year | **Urban** | **Rural** |
| 1999 | 0.4 (0.4–0.4) | 0.2 (0.2–0.3) |
| 2000 | 0.6 (0.5–0.6) | 0.4 (0.3–0.4) |
| 2001 | 0.6 (0.6–0.7) | 0.5 (0.4–0.5) |
| 2002 | 0.7 (0.6–0.7) | 0.6 (0.5–0.6) |
| 2003 | 0.7 (0.7–0.8) | 0.6 (0.6–0.7) |
| 2004 | 0.7 (0.7–0.8) | 0.7 (0.6–0.7) |
| 2005 | 0.8 (0.8–0.9) | 0.8 (0.7–0.8) |
| 2006 | 0.9 (0.8–0.9) | 0.9 (0.8–0.9) |
| 2007 | 0.6 (0.6–0.7) | 0.6 (0.6–0.7) |
| 2008 | 0.7 (0.6–0.7) | 0.7 (0.6–0.7) |
| 2009 | 0.7 (0.7–0.7) | 0.7 (0.7–0.8) |
| 2010 | 0.8 (0.7–0.8) | 0.7 (0.7–0.8) |
| 2011 | 0.8 (0.8–0.9) | 0.9 (0.8–1.0) |
| 2012 | 0.9 (0.8–0.9) | 1.0 (0.9–1.1) |
| 2013 | 0.9 (0.9–0.9) | 1.1 (1.0–1.2) |
| 2014 | 1.0 (1.0–1.0) | 1.2 (1.1–1.3) |
| 2015 | 1.0 (1.0–1.0) | 1.3 (1.2–1.4) |
| 2016 | 1.0 (1.0–1.0) | 1.3 (1.2–1.4) |
| 2017 | 1.0 (1.0–1.0) | 1.3 (1.2–1.4) |
| 2018 | 1.0 (0.9–1.0) | 1.3 (1.2–1.4) |
| 2019 | 0.9 (0.9–1.0) | 1.3 (1.2–1.3) |
| 2020 | 1.0 (0.9–1.0) | 1.5 (1.4–1.6) |
| 2021 | 0.6 (N/A–N/A) | 0.8 (N/A–N/A) |
| 2022 | 0.6 (N/A–N/A) | 0.8 (N/A–N/A) |
| 2023 | 0.6 (N/A–N/A) | 0.8 (N/A–N/A) |
| Overall | 0.80 (0.79–0.82) | 0.89 (0.87–0.91) |
